# Genetic and pharmacological inhibition of Cdk1 provides neuroprotection towards ischemic neuronal death

**DOI:** 10.1038/s41420-018-0044-7

**Published:** 2018-03-16

**Authors:** Quentin Marlier, Florian Jibassia, Sébastien Verteneuil, Jérôme Linden, Philipp Kaldis, Laurent Meijer, Laurent Nguyen, Renaud Vandenbosch, Brigitte Malgrange

**Affiliations:** 1 Laboratory of Developmental Neurobiology, GIGA-Neurosciences, University of Liège, C.H.U. B36, 4000 Liège, Belgium; 2 Department of Psychology, University of Liege, B32, 4000 Liège, Belgium; 3 Institute of Molecular and Cell Biology (IMCB), A*STAR (Agency for Science, Technology and Research), 61 Biopolis Drive, Proteos#3-09, Singapore, 138673 Republic of Singapore; 4 Department of Biochemistry, National University of Singapore (NUS), Singapore, 117597 Republic of Singapore; 5 ManRos Therapeutics, Centre de Perharidy, 29680 Roscoff, France; 6 Laboratory of Molecular Regulation of Neurogenesis, GIGA-Neurosciences, University of Liège, C.H.U. B36, 4000 Liège, Belgium

## Abstract

Cell cycle proteins are mainly expressed by dividing cells. However, it is well established that these molecules play additional non-canonical activities in several cell death contexts. Increasing evidence shows expression of cell cycle regulating proteins in post-mitotic cells, including mature neurons, following neuronal insult. Several cyclin-dependent kinases (Cdks) have already been shown to mediate ischemic neuronal death but Cdk1, a major cell cycle G2/M regulator, has not been investigated in this context. We therefore examined the role of Cdk1 in neuronal cell death following cerebral ischemia, using both in vitro and in vivo genetic and pharmacological approaches. Exposure of primary cortical neurons cultures to 4 h of oxygen–glucose deprivation (OGD) resulted in neuronal cell death and induced Cdk1 expression. Neurons from Cdk1-cKO mice showed partial resistance to OGD-induced neuronal cell death. Addition of R-roscovitine to the culture medium conferred neuroprotection against OGD-induced neuronal death. Transient 1-h occlusion of the cerebral artery (MCAO) also leads to Cdk1 expression and activation. Cdk1-cKO mice displayed partial resistance to transient 1-h MCAO. Moreover, systemic delivery of R-roscovitine was neuroprotective following transient 1-h MCAO. This study demonstrates that promising neuroprotective therapies can be considered through inhibition of the cell cycle machinery and particularly through pharmacological inhibition of Cdk1.

## Introduction

Despite several neuroprotective clinical trials, protecting the brain against ischemic injury, the second leading cause of death affecting one in every six people worldwide, remains an unsolved and challenging question. Administration of tissues plasminogen activator is currently the only treatment used to reduce damage from stroke with medium efficacy^1^. Understanding the molecular mechanisms underlying stroke pathophysiology is critical to develop more effective therapies.

Following ischemia, neurons undergo irreversible damage in the ischemic necrotic core within minutes or few hours. Close to the occluded artery, the blood flow decreases by >80%, depriving neurons of oxygen and glucose and leading to the disruption of the ionic gradient, increased intracellular Ca^2+^ concentration, membrane degradation and cell swelling. Surrounding the ischemic core, the moderately perfused peri-infarct area undergoes delayed neuronal death within hours or days, via excitotoxicity^2^ or other mechanisms. This delayed neuronal loss represents a therapeutic time window for neuroprotective strategies. This has not yet been translated into clinical trials. The lack of understanding regarding the intrinsic signaling pathways involved in neuronal apoptotic death may be a critical factor, which contributes to translational therapy failures. An important network of death signals regulates ischemic neuronal death but most of them remain poorly understood. Therefore, there is a need to investigate the molecular mechanisms and identify potential drug targets to develop therapies able to reduce stroke-related brain damage.

Core cell cycle serine/threonine kinases, cyclin-dependent kinases (Cdks), are Cdk1, Cdk2, Cdk4 and Cdk6 and are expressed in dividing cells, whereas Cdk5, an atypical Cdk, is expressed mainly in post-mitotic cells, including neurons. Inappropriate re-expression of core cell cycle Cdks and Cdk5 overactivation in neurons leads to cell death. Indeed, besides their role in cell cycle control, Cdks have been reported as potential mediators of ischemic neuronal death^3^ and involved in neurodegenerative diseases^4^. However, the role of Cdk1 and its effect on stroke outcomes are not known.

Cdk1 is the mitotic member of the cell cycle family. Following cyclin A or B binding, Cdk1 is activated and phosphorylates key substrates allowing the cell to complete G2-phase, enter M-phase and proceed to cytokinesis. A link between Cdk1 and apoptotic cell death has already been shown^5^. Moreover, Cdk1 and cyclin B1 are expressed in the cortex of mice following transient ischemia^6^. Therefore, alleviating Cdk1 in neurons may represent an effective strategy to preserve neuronal integrity in the peri-infarct area.

In this study, we used both genetic and pharmacological approaches to investigate the role of Cdk1 following ischemia. We demonstrate that Cdk1 is re-expressed in neurons deprived of oxygen and glucose (oxygen–glucose deprivation (OGD) model) and that the lack of Cdk1 in cortical neuronal cultures drastically increases neuronal survival/death ratio following OGD. Interestingly, this in vitro neuroprotective effect is reproduced using R-roscovitine. Using a mouse model of human cerebral ischemic damage, we confirmed these results in vivo. Indeed, following middle cerebral artery occlusion (MCAO), Cdk1 re-expression and activation is observed in the post-ischemic mouse cortex. In addition, a significant reduction of the damaged area is seen in Cdk1-cKO or R-roscovitine-treated mice. Altogether, our findings highlight the role of the G2/M-phase cell cycle regulator Cdk1 in neuronal death following ischemia.

## Results

### OGD induces caspase-3 activation and Cdk1 expression in vitro

**Fig. 1:**
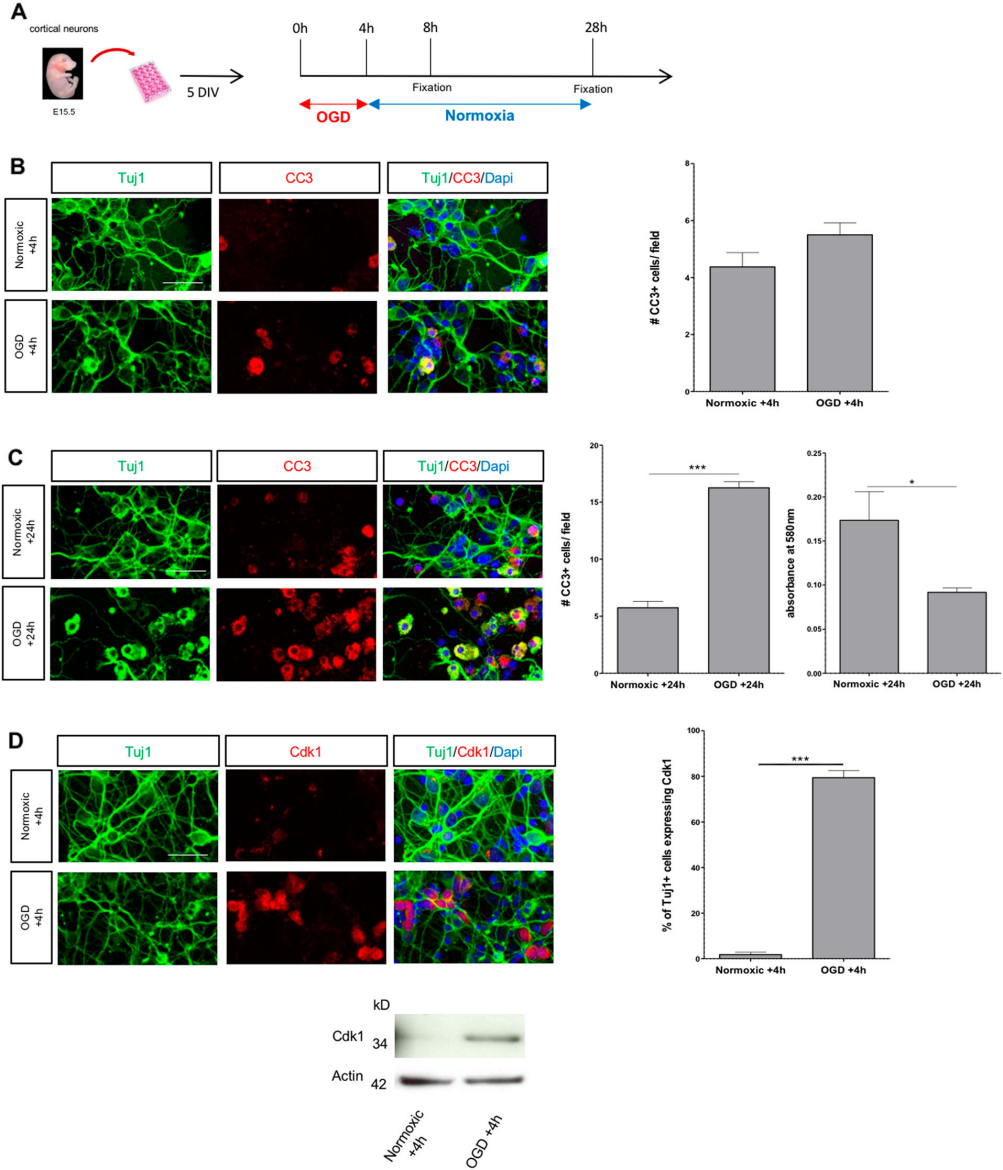
OGD induces caspase-3 activation and Cdk1 expression. **a** Scheme of the experimental protocol. **b** Representative confocal microscopy images and quantitative results showing no differences in the number of CC3-positive cells between normoxic and OGD condition 4 h following OGD, scale bar = 30 µm and field size = 120 µm × 70 µm (data represent means ± SEM; *n* = 4; ns; Student’s *t*-test). **c** Representative confocal microscopy images and quantitative results illustrating significant differences in the number of CC3-positive cells (data represent means ± SEM;* n* = 4; ***p < 0.001; Student’s *t*-test) and survival (MTT assay) (data represent means ± SEM; *n* = 5; *p < 0.01; Student’s *t*-test) between normoxic and OGD condition at 24 h following OGD. **d** Representative confocal images showing OGD-induced Cdk1 expression 4 h following OGD compared with normoxic condition, scale bar = 30 µm. Quantitative results represent the percentage of living (CC3-negative) neurons expressing Cdk1, field size = 120 µm × 70 µm (data represent means ± SEM; *n* = 5; ***p < 0.001; Student’s *t*-test). Western blot analysis demonstrated increased Cdk1 expression 4 h following OGD

To investigate the role of Cdk1 in ischemic neuronal death, we used an in vitro model of ischemia–reperfusion. After 5 days in vitro (DIV), we exposed E15.5 mouse cortical neurons to OGD and then quantified neuronal cell death 4 or 24 h later by immunolabeling with antibodies against cleaved caspase-3 (CC3) and beta-III tubulin (Tuj1) (Fig. 1a–c). Although no difference of cell survival was observed at 4 h between conditions (Fig. 1b), incubation in OGD medium for 24 h led to increase in neuronal death, as compare with control. Reduced cell survival was further confirmed by 3-(4,5-dimethylthiazol-2-yl)-2,5-diphenyl tetrazolium bromide (MTT) assay (Fig. 1c). We then investigated the expression of Cdk1 after 24 h of culture in OGD condition. Although no Cdk1 was immunodetected in cortical neurons in normoxic conditions, nuclear expression of Cdk1 was observed 4 h post-OGD (Fig. 1d).

### Lack of Cdk1 or Cdk1 inhibition protects against OGD-induced neuronal death in vitro

Mice bearing a Cdk1^lox^ allele were crossed with Nex^Cre/+^ transgenic mice to specifically delete Cdk1 in glutamatergic post-mitotic neurons of the cortex and hippocampus (thereafter termed Cdk1-cKO mice), without affecting proliferating precursors. We first assessed whether Cdk1 affects neuronal development and survival under physiological conditions. At P30, brain weight, as well as cortical thickness and neuronal number, were comparable between Ctrl and Cdk1-cKO both in rostral and medial cortical regions (Supplementary Figure 1), suggesting that Cdk1 is dispensable for neuronal survival during development under these conditions.

**Fig. 2:**
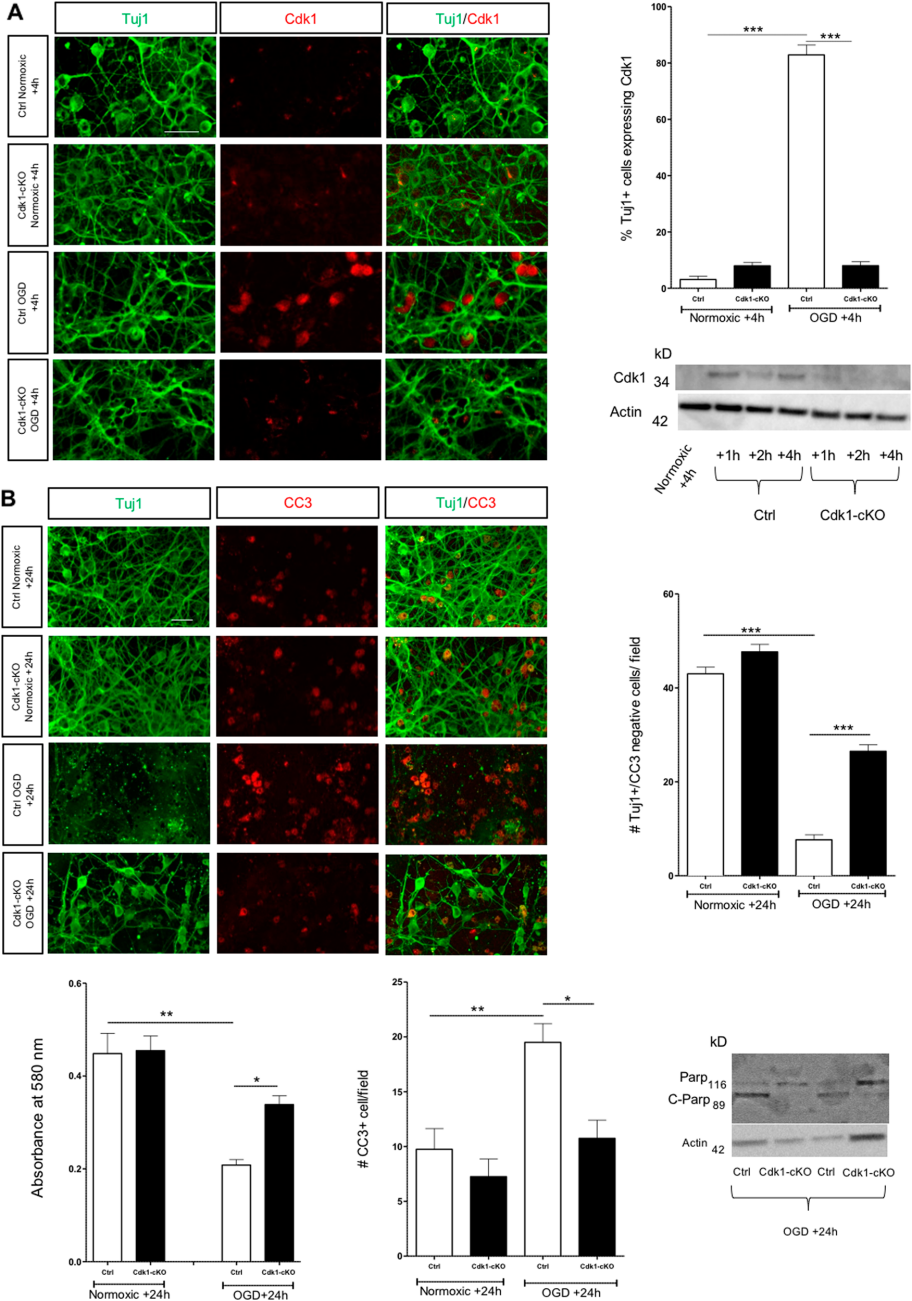
Loss of Cdk1 protects neurons against OGD-induced death. **a** Representative confocal images illustrating OGD-induced Cdk1 neuronal expression, scale bar = 30 µm. Quantitative results represent the percentage of Tuj1-positive living neurons (green) expressing Cdk1 (red), field size = 140 µm × 90 µm (data represent means ± SEM; *n* = 4; ***p < 0.001; ANOVA with Bonferroni’s post hoc). Western blot analysis indicated increased Cdk1 expression 1, 2 and 4 h following OGD. **b** Representative confocal images illustrating the number of Tuj1-positive (green) and CC3-positive (red) cells in the different conditions/genotypes 24 h following OGD, scale bar = 30 µm. Quantitative results for neuronal survival represents the number of Tuj1-positive/CC3-negative cells, field size = 230 µm × 150 µm (data represent means ± SEM; *n* = 5; ***p < 0.001; one-way ANOVA with Bonferroni’s post hoc). MTT assay confirm significant difference regarding neuronal survival between the different conditions 24 h following OGD (data represent means ± SEM; *n* = 5; *p<0.05; **p<0.01 ; one-way ANOVA with Bonferroni’s post hoc). Quantitative results for neuronal death represent the number of CC3-positive cells, field size = 230 µm × 150 µm (data represent means ± SEM; *n* = 4; *p<0.05; **p < 0.005; one-way ANOVA). Western blot analysis showed decreased C-Parp (89 kDa) protein expression in Cdk1-cKO OGD-treated cultures as compared with Ctrl OGD-treated cultures

To test whether Cdk1 expression underlies OGD-induced neuronal death, we cultured cortical neurons from E15.5 Ctrl and Cdk1-cKO embryos. We first validated our model by assessing Cdk1 expression. Whereas Cdk1 was re-expressed 4 h after OGD treatment in Ctrl cortical neurons, it was mostly absent in normoxic condition, as well as in Cdk1-cKO neurons both in normoxic and OGD conditions (Fig. 2a). As Nex is only expressed in glutamatergic neurons, Cdk1 expression found in Cdk1-cKO cultures likely comes from GABAergic neurons or from contaminating glial cells, representing <5% of cultured cells (Supplementary Figure 2).

We next analyzed the effect of the conditional deletion of Cdk1 on projection neurons viability by combining immunolabeling for CC3-positive neurons and by performing MTT bioassay. Although neuronal survival rates were comparable between Ctrl and Cdk1-cKO cultures under normoxic conditions, Cdk1-cKO neuronal cultures displayed greater survival and less apoptosis as compare with Ctrl neuronal cultures, 24 h post-OGD treatment (Fig. 2b). Similarly, western blot analysis of cleaved Poly(ADP-ribose) polymerase (PARP) (C-parp), a late apoptosis marker, revealed that the absence of Cdk1 protected neuronal cells against OGD-induced neuronal death (Fig. 2b), as the levels of C-parp were much lower than in control.

**Fig. 3:**
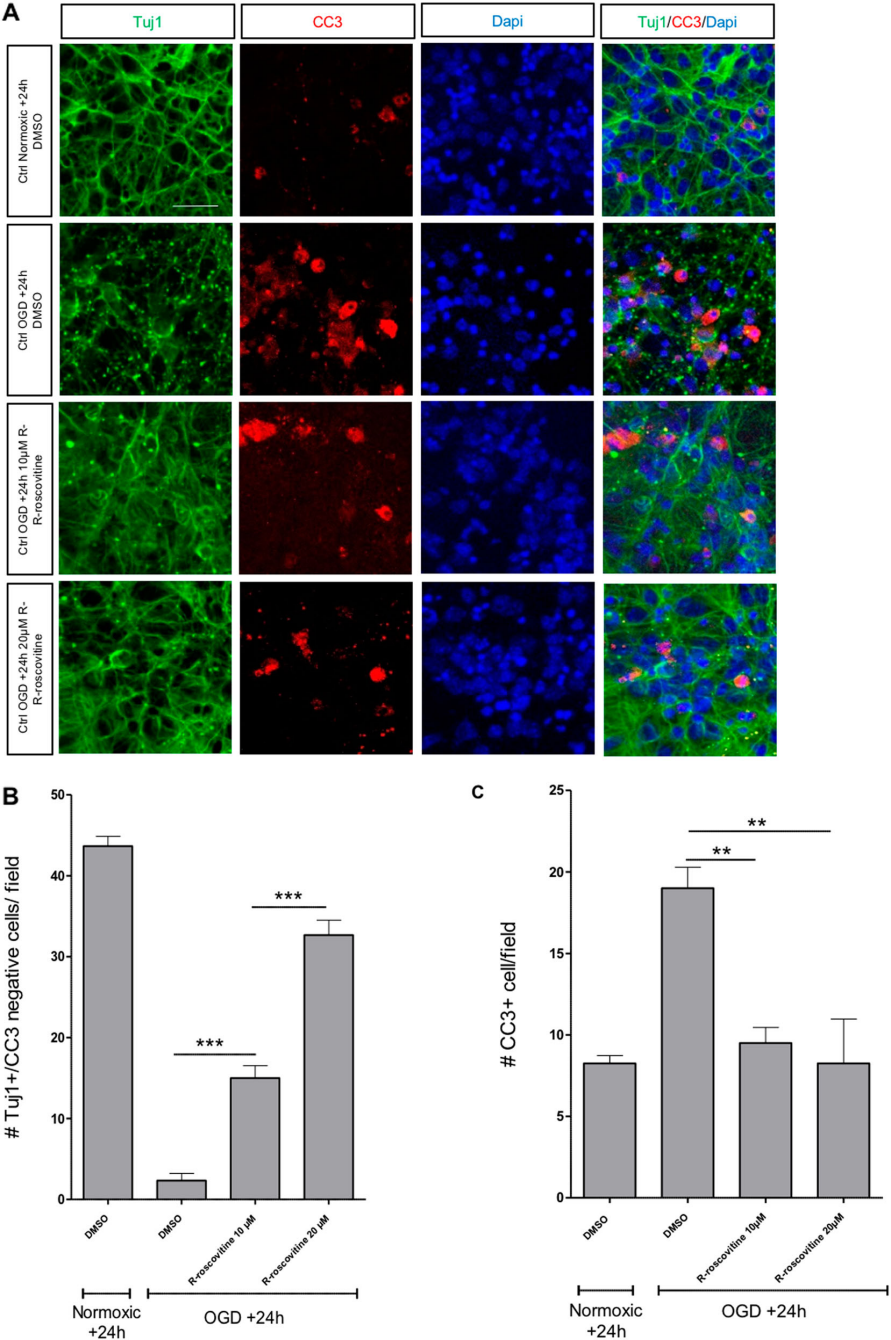
Treatment with the Cdk inhibitor R-roscovitine protects neurons against OGD-induced neuronal death. **a** Representative confocal images illustrating normoxic and OGD neurons treated with DMSO or R-roscovitine (10 µM or 20 µM), showing the dose-dependent protective effect of R-roscovitine against OGD, scale bar = 30 µm. **b** Neuronal survival represented by the number of Tuj1-positive/CC3-negative cells, field size = 114 µm × 125 µm (data represent means ± SEM; *n* = 4; ***p < 0.001; one-way ANOVA with Bonferroni’s post hoc) and **c** quantitative results represent the number of apoptotic CC3-positive neurons, field size = 114 µm × 125 µm (data represent means ± SEM; *n* = 4; **p < 0.005; one-way ANOVA with Bonferroni’s post hoc)

To confirm these results, we treated cortical cultures with R-roscovitine, a well-characterized pharmacological inhibitor of Cdk1 (ref. ^7^), which also inhibits Cdk2, Cdk5, Cdk7 and Cdk9. The number of surviving neurons 24-h post-OGD was significantly increased in the presence of R-roscovitine at 10 and 20 µM (Fig. 3a, b), whereas higher concentrations were toxic (data not shown). In addition, we observed that the presence of R-roscovitine significantly decreased the percentage of CC3-positive cells 24-h post-OGD (Fig. 3c) to the levels seen under normoxic conditions. Taken together, these results indicate that: (1) Cdk1 expression tightly correlates with neuronal apoptotic death after in vitro OGD; (2) reduced expression (Cdk1-cKO) or reduced catalytic activity of Cdk1 by pharmacological inhibition (R-roscovitine treatment) provides neuroprotection, further suggesting a link between Cdk1 activity and the apoptotic cell death resulting from OGD treatment.

### Transient cerebral ischemia induces Cdk1 expression and activation

**Fig. 4:**
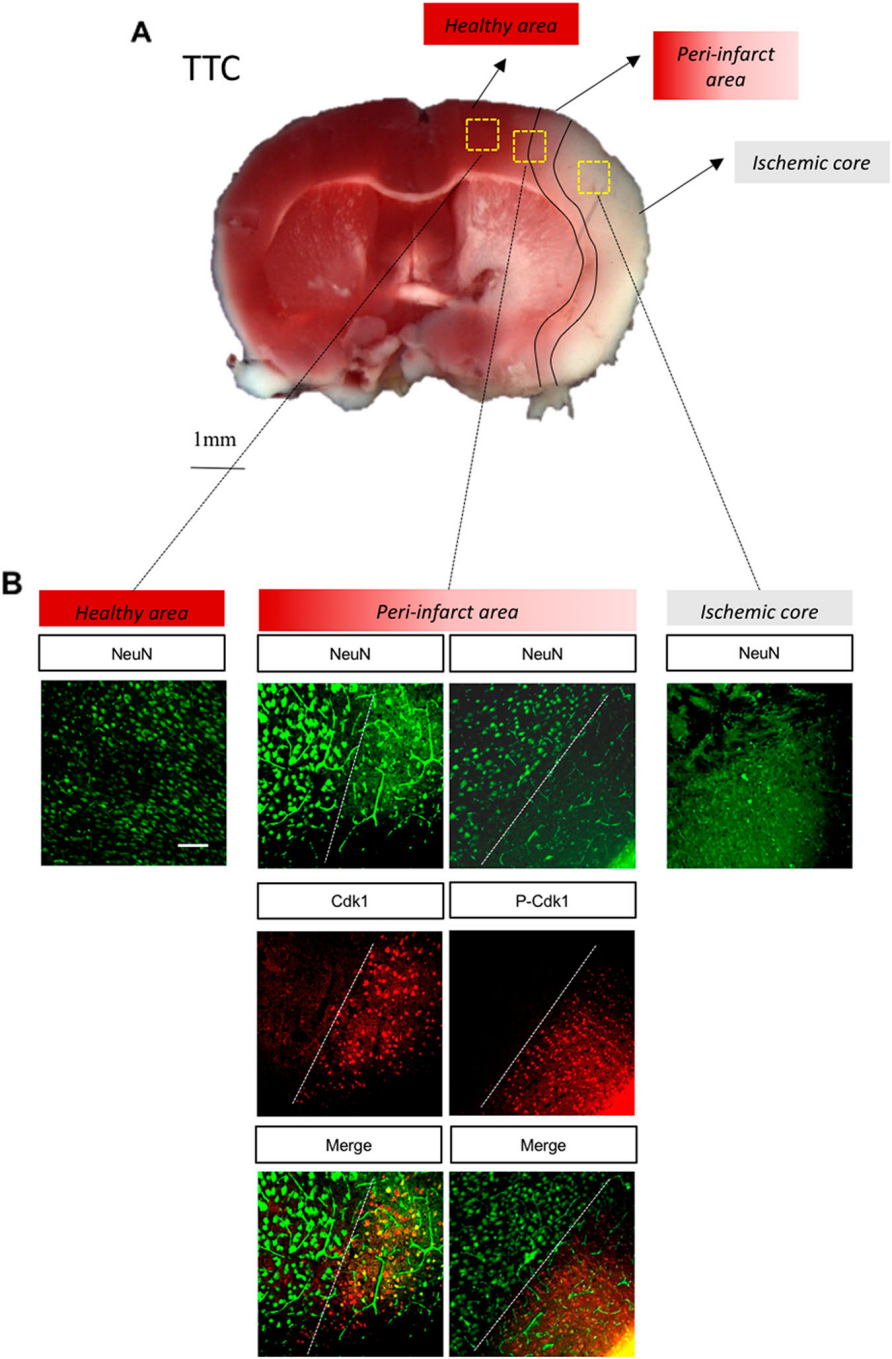
Transient cerebral ischemia induces Cdk1 expression and activation. **a** Photography of 2 mm thick TTC-stained mouse brain coronal section 24 h after a transient 1 h MCAO, showing the healthy cortex (red), ischemic core (white) and peri-infarct area (between red and white) in the ipsilateral cortex. **b** Immunohistochemistry images showing NeuN staining in the healthy part of the ipsilateral cortex and the absence of NeuN staining in the ischemic core. In between, the peri-infarct area shows re-expression of Cdk1 and P-Cdk1. Scale bar = 100 µm

The role of Cdk1 in ischemic neuronal death was further investigated in vivo using a temporary MCAO mouse model. WT mice were subjected to transient MCAO for 1 h followed by 24-h reperfusion. 2,3,5-Triphenyltetrazolium chloride (TTC) staining allowed the identification of the healthy area (red), the ischemic core (white) and, in between, the peri-infarct area (Fig. 4a). Immunohistochemistry analysis of the ipsilateral ischemic core indicated an extensive downregulation of NeuN-positive neurons, in contrast to the healthy non-ischemic area (Fig. 4b). Consistent with the in vitro results, Cdk1, as well as its Thr161-phosphorylated form (p-Cdk1), was not present in the healthy area 24-h post-MCAO, whereas it appears to be highly expressed in the peri-infarct area (Fig. 4b). These results suggest that Cdk1 may be involved in neuronal apoptotic death after in vivo ischemia.

### Lack of Cdk1 or R-roscovitine treatment protects against ischemic-induced neuronal death in vivo

**Fig. 5:**
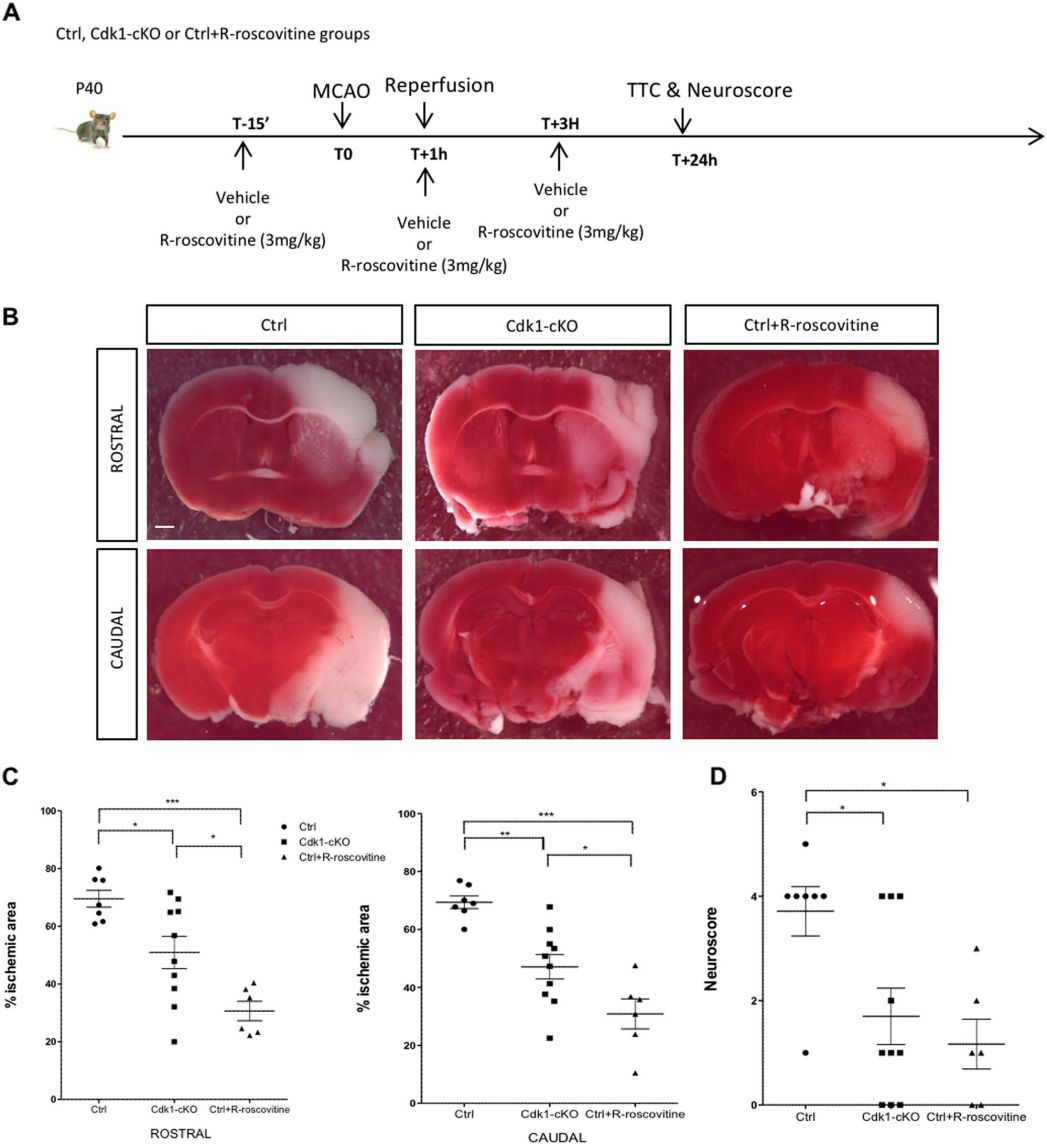
Lack of Cdk1 or R-roscovitine treatment protects against ischemic-induced neuronal death in vivo. **a** Scheme of the experimental protocol. **b** TTC-stained rostral and caudal brain sections from vehicle-treated Ctrl and Cdk1-cKO mice and R-roscovitine-treated Ctrl mice after 1-h MCAO and 24 h of reperfusion. Scale bar = 1 mm. **c** Rostral and caudal quantification of infarct size (% brain area) (data represent means ± SEM; *n* = 6-10; *p<0.05; **p<0.005; ***p < 0.001; ANOVA with Bonferroni’s post hoc). **d** Quantification of neuroscore scale (data represents means ± SEM; *n* = 6–10; *p < 0.01; ANOVA with Bonferroni’s post hoc)

We then investigated the consequences of the lack of Cdk1 or its pharmacological inhibition of catalytic activity on neuronal survival in vivo following MCAO. For this purpose, Ctrl or Cdk1-cKO mice were subjected to transient MCAO for 1 h followed by 24-h reperfusion. Cdk1-cKO mice showed a smaller cerebral infarct area both at a rostral or caudal position as compared with the WT mice (Fig. 5b, c and Supplementary Figure 3). Moreover, neuroscore results suggested that Cdk1-cKO mice demonstrated remarkably higher recovery of motor function than the WT mice (Fig. 5d). Altogether, these data provide evidence that loss of Cdk1 attenuates ischemic brain damage. To confirm these results, we investigated whether pharmacological inhibition of Cdk1 also ameliorates cerebral ischemia–reperfusion injury. Control mice received three injections of R-roscovitine (3 mg/kg/i.p.) 15 min before, 1 h and 3 h after stroke. As shown in Fig. 5b, c, the size of ischemic area is significantly diminished in mice treated with R-roscovitine as compared with controls. Moreover, R-roscovitine treatment ameliorated the neurological deficit score (Fig. 5d). Collectively, these data suggest that Cdk1 is a key mediator of neuronal loss following stroke.

## Discussion

Increasing evidence suggests that cell cycle machinery is strongly involved in stroke-induced neuronal death^3, 8^. Indeed, the expression of cyclin D, Cdk4 and their specific targets E2F and pRB is increased following stroke or ischemic insult^8,9^. Their respective inhibition or downregulation has been shown to be neuroprotective from ischemia-induced cell death both in vitro and in vivo^10,11^. Late G1/S-phase cell cycle proteins, such as Cdk2 and cyclin E, have also been detected following ischemia^9,12,13^. Finally, although there is little knowledge regarding cell cycle regulators in human stroke, a study has reported elevated levels of numerous cell cycle regulatory proteins in human brains following cardiac arrest or focal brain infarction^14^. These proteins include cyclins (A, D1 and E) and Cdks (2 and 4). Hence, some cell cycle regulators potentially represent a group of interesting therapeutic targets for the development of stroke drug candidates.

In the present study, we demonstrated that Cdk1 is an in vitro and in vivo key mediator of ischemic-induced neuronal death. Indeed, we observed neuroprotective effects of genetic elimination of Cdk1 in hypoxic neurons in culture and in the ischemic cortex. Interestingly, we confirmed these results using R-roscovitine, a pharmacological blocker of Cdk1, Cdk2, Cdk5, Cdk7, Cdk9 and casein kinases 1 (CK1). A recent study has already shown that S-roscovitine (R-roscovitine is the isomer, which is in clinical trials, reviewed in Meijer et al.^15^) is neuroprotective following ischemia^16^. This neuroprotective effect of S-roscovitine was proposed to be, at least partially, Cdk5 dependent^17^. This Cdk5 inhibition effect was confirmed by another study in rat^18^. Cdk5 is a “non-cell cycle” Cdk, whose activation requires the association with neuron-specific proteins, p39 or p35. Following in vitro or in vivo neuronal ischemia, excitotoxic release of glutamate induces a Ca^2+^ overload that consequently activates calpain. Key substrates of calpain are p39 and p35, which are, respectively, converted to p29 and p25 (ref. ^19^). The p25 fragment triggers Cdk5 hyperactivation that consequently induces phosphorylation of N-methyl-D-aspartate (NMDA) receptors, synaptic dysfunction, cell cycle re-entry, mitochondrial dysfunction and cellular apoptosis^20^. The effect of R-roscovitine treatment on MCAO is probably accounted for inhibitory effects on both cell cycle Cdks (i.e., Cdk1 and/or 2) and the non-cell cycle Cdk5. Coherent with this hypothesis is the statistically significant higher level of neuroprotection seen in R-roscovitine-treated mice compared with Cdk1-cKO mice (Fig. 5). Therefore, downregulation of one Cdk alone would not be sufficient to reach significant clinical outcome, a limited problem since Cdk1, Cdk2 and Cdk5 inhibition by pharmacological inhibitors are essentially tightly connected. However, we cannot exclude the possibility that this difference is due to the cell type-specific Cre recombination occurring in our genetic model, in which only glutamatergic neurons are affected. For instance, GABAergic inhibitory neurons represent around 20% of the neuronal population in the adult cortex^21^. Finally, CK1s—in particular the brain CK1ε—also represent a significant target of roscovitine^22^, and it is possible that inhibition of CK1s may contribute to the beneficial effects of Cdk1/2/5 inhibition in stroke models.

Cdk5 has a fundamental role in the physiology of post-mitotic neurons^23^. Moreover, cyclin E is able to form complex with Cdk5 to regulate synaptic and memory formation^24^. As a consequence, long-term inhibition of Cdk5 for stroke treatment may induce major side effects. In addition, increased Cdk5 expression has been detected in endothelial cells and might exert neuroprotective effect by promoting angiogenesis to increase reperfusion following cerebral ischemia^25^. Therefore, a solution to avoid deleterious effects of p35/Cdk5 complexes inhibition could be to specifically target p25/Cdk5 and p29/Cdk5 complexes. Indeed, it has been shown that an inhibitory peptide of the p25/Cdk5 complex was able to reduce neurodegeneration in vivo without affecting normal p35/Cdk5 activity^26^. Taken together, the combination of this peptide with a specific Cdk1 inhibitor—such as RO-3366—may lead to an efficient neuroprotection and stroke recovery.

How Cdk1 promotes neuronal death remains to be determined. In multiple neuronal death paradigms, activation of Cdk1 has been associated with phosphorylation of the Bcl-2 family of proteins. Indeed, Cdk1 is able to induce activating phosphorylation of the pro-apoptotic protein Bad^27^ or inhibitory phosphorylation of anti-apoptotic Bcl-xL, Bcl-2 and Mcl-1 proteins^28,29^. Moreover, Cdk inhibition by R-roscovitine (and other inhibitors) leads to a reduction of Mcl-1 (ref. ^30^). Besides the Bcl-2 family, Cdk1 has also been found to be able to phosphorylate the transcription factor FOXO1, which promotes cell death signaling pathways^31^. Further studies need to be performed in order to determine if those molecular pathways are involved in Cdk1-induced ischemic neuronal death.

Interestingly, synergistic neuroprotective effect of Cdk1 and Cdk5 inhibition may be extended to other neuropathological conditions, as both enzymes are individually recognized to contribute to cell death in several neurologic diseases. Pharmacological or genetic inhibition of Cdk5 offers protection against neuronal death in Alzheimer’s disease (AD)^32^ and Parkinson’s disease (PD)^33^ models. Numerous pieces of evidence also suggest that Cdk1 is involved in neuronal death following spinal cord injury^34^ or AD^35^.

Taken together, our data demonstrate that Cdk1 contributes to ischemic-induced neuronal death. However, other Cdks—both cell cycle-related and not—should also be targeted to increase neuronal survival rate. R-roscovitine, which has undergone many clinical trials (over 525 patients), should therefore be considered in clinical trials for stroke.

## Materials and methods

### Animals

All mice were maintained on a pure C57BL/6J background. Wild-type mice were used to study caspase-3 activation in vitro and Cdk1 expression level in vitro and in vivo. To study the consequences of loss of Cdk1 on neuronal death, Cdk1^lox/lox^ mice (Dr. P. Kaldis, IMCB, Singapore)^36^ were crossed with Nex^Cre/+^ mice (Dr. K.A. Nave, Max Planck Institute of Experimental Medicine, Gottingen, Germany)^37^ to obtain Cdk1^lox/lox^ (Ctrl) and Cdk1^lox/lox^;Nex^Cre/+^ (Cdk1-cKO) mice. The genotype was determined by polymerase chain reaction (PCR) as previously described^36,37^. Mice were group housed in the animal facility at the University of Liège under standard conditions with food and water ad libitum and were maintained on a 12-h light/dark cycle. All animals were taken care of in accordance with the Declaration of Helsinki and following the guidelines of the Belgian Ministry of Agriculture in agreement with EC laboratory animal care and use regulation (2010/63/UE, 22 September 2010). All experiments were approved by the Animal Care Ethics Committee of the University of Liège (protocol N°1694).

### Primary cortical neuronal culture

E15.5 pregnant female mice were humanely euthanized and embryos removed. Cerebral cortices were then microdissected and dissociated into reduced serum medium (Opti-MEM^TM^, ThermoFisher) using enzymatic (trypsin-DNase solution) and mechanical dissociation. Cultures plates were coated with 40 µg/ml poly-l-lysine (Merck-Sigma, #P7280) and 6 µg/ml laminin (Merck-Sigma, #L2020) and cells were cultured in Neurobasal medium (ThermoFisher, #21103049), supplemented with 2% B-27 supplement (ThermoFisher, #17504044), 1% l-glutamine (Lonza, #17-605 C), and 1% penicillin/streptomycin (ThermoFisher, #10378016). Primary cortical neurons were maintained for 5 days in a humidified incubator at 37 °C with 5% CO_2_ before experiments started. Neuronal enrichement was assessed by counting the number of Tuj1-positive and Glial fibrillary acidic protein (GFAP)-positive cells. Limited astroglial contamination (±5%) with GFAP-positive cells was observed in our culture conditions (Supplementary Figure 2).

### Oxygen–glucose deprivation

Ischemia-like conditions were obtained by omitting glucose in the culture medium at 5 DIV with deoxygenated glucose-free Earle’s balanced salt solution (Borate buffer saline; 0.01 mM glycine pH 7.4, 120 mM NaCl, 5 mM KCl, 2 mM MgSO_4_, 1.25 mM NaH_2_PO_4_, 25 mM NaHCO_3_, 2 mM CaCl_2_) and placing cells in an anaerobic chamber (Heracell^TM^ 150, Thermo Scientific, Waltham, MA, USA) containing a gas mixture of 5% CO_2_, 1% O_2_. After 4 h, OGD was terminated by returning the cultures to normoxic conditions and neurobasal medium supplemented with 2% B-27 supplement, 1% l-glutamine and 1% penicillin/streptomycin.

### R-roscovitine treatment

R-roscovitine was obtained from ManRos Therapeutics. For in vitro experiments, R-roscovitine was dissolved in 0.1% dimethylsulfoxide (DMSO) and was used at final concentrations of 10, 20 or 50 µM. R-roscovitine was added to the culture media at DIV 5 during OGD (4 h), whereas 0.1 % DMSO was applied to control cultures for the same time. For in vivo experiments, R-roscovitine was dissolved in 5% DMSO, 50% polyethylene glycol (PEG300), 45% H_2_O and was used at a final concentration of 0.3 mg/mL.R-roscovitine was injected (intraperitoneally) at 3mg/kg 15 min before surgery, 1- and 3-h post-surgery. R-roscovitine was stored at −20 °C for long time storage and at +4 °C during the experiments.

### Immunostaining

Sections or cultures were fixed by replacing the culture medium with 4% paraformaldehyde (4.3 g/L NaOH; 40 g/L Paraformaldehyde (PFA); 18.8 g/L NaH_2_PO_4_) for 10 min. Sections were then transferred to 20% sucrose until equilibration and frozen in tissue-Tek OCT (Tissue OCT, VWR # 00411243) to allow cutting of 40 µm coronal sections placed in phosphate-buffered saline (PBS)/azide (0.1%). Coverslips or sections were then incubated overnight in PBS containing Tween (0.1%), Triton (0.1%) with 5% donkey serum and primary antibody. The following antibodies were used at the indicated dilutions: rabbit monoclonal anti-β-III tubulin (Tuj1) (1:500; BioLegend cat# 801202, RRID:AB_10063408), rabbit polyclonal anti-phospho-Cdk1 (Thr161) (1:1000; Cell Signaling Technology cat# 9111S, RRID:AB_331460), rabbit anti-Cdk1 (1:500; Atlas Antibodies cat# HPA003387, RRID:AB_1846356), rabbit anti-CC3 (1:1000; Cell Signaling Technology cat# 9661, RRID:AB_2341188), mouse anti-GFAP (1:500; Sigma-Aldrich cat# G3893, RRID:AB_477010), mouse monoclonal anti-Cdk1 (1:500; BD Biosciences cat# 612306, RRID:AB_399621), mouse anti-NeuN (1:500, Millipore cat# MAB377, RRID:AB_2298772). Coverslips or sections were then washed in PBS before incubated 1 h at room temperature in PBS containing Tween (0.1%), Triton (0.1%) and the corresponding donkey-raised secondary antibodies conjugated to Alexa Fluor 488 or 555 (ThermoFisher). Finally, coverslips or sections were washed in PBS and mounted in VectaShield Hard Set medium containing DAPI (Vector Laboratories). Images were collected using a confocal microscope (Nikon A1 system) and quantitative analysis was performed by counting three 40× random fields per coverslip. NeuN immunolabeling also allowed us to determine the ischemic boundary area characterized by a sharp reduction of its staining, which is of key importance for the final stroke outcome^38^.

### Western blotting

Primary cultures at 5, 6, 8 and 28 h were lysed on ice in a solution containing 50 mM Tris-HCl pH 8, 60 mM sodium glycerophosphate, 150 mM NaCl, 10 mM disodium phenylphosphate, 500 mM NaF, 100 mM Na_3_VO_4_, 1% NP40 and protease inhibitors (Complete Mini, EDTA-Free, Sigma-Aldrich, # 11836170001). Protein concentration was determined using Bio-Rad Protein Assay (Bio-Rad). Proteins (30 µg per lane) were separated on 4–12% Bis-Tris Plus gels (Invitrogen) and transferred to Polyvinylidene difluoride (PVDF) immunoblotting membrane (Millipore). The membranes were blocked with 5% milk in Triton-Tween 20 buffer solution (TTBS) for 1 h and then incubated in 5% milk/TTBS with primary antibody overnight. Membranes were probed with rabbit anti-Cdk1 (1:500; Atlas Antibodies cat# HPA003387, RRID:AB_1846356) and rabbit anti-cleaved Parp (Asp214) (1:1000; Cell Signaling Technology cat# 9541, RRID:AB_331426) and anti-β-actin peroxidase conjugated (1:25000; Sigma-Aldrich cat# A3854, RRID:AB_262011). Immunoreactive bands were detected using donkey anti-rabbit secondary antibodies conjugated to peroxidase (1:10,000; Abcam). Pierce ECL Western Blotting substrate (ThermoFisher, #32106) was used to detect immunoreactive bands.

### MTT assay

After 28 h of culture, the medium was removed from the 96-well plates and 50 µL of 1.5 mg/mL MTT (Sigma-Aldrich, #CT01-5) solution in Minimum essential media (MEM) were added for 3 h. After removal of the MTT solution, isopropanol-HCl (20 mL/120 µL) solution was added to each well to dissolve the formazan crystals accumulated within living cells. The absorbance at 540 nm was measured using Labsystems Multiskan reader (LabX).

### Transient MCAO

Adult male mice, randomly assigned to the experiment groups, were anesthetized with isoflurane (IsoFlo, Zoetis #B506) and body temperature was maintained at 37 °C with a heating pad. A midline incision was performed in order to expose the right common carotid artery. The common carotid artery and the external carotid artery were ligated with a 5-0 silk suture. A third ligature was performed on the common carotid artery, at the level of the bifurcation of the external and internal carotid artery. A slight hole was made between both holes in the common carotid artery to allow insertion of a 6-0 coated monofilament (Doccol), which was moved from the common carotid artery through the internal carotid artery up to the level of the middle cerebral artery (10–11 mm). After 1 h of MCAO, the monofilament was withdrawn to allow reperfusion. The number of animals used for analysis or excluded are listed in Table 1. A formal sample size and power calculations were not performed because this was the first investigation using this protocol.

**Table 1:** Number of used/excluded animals

Experiment		Total (*n*)	Exclusion		Final (*n*)	Mean weight (g)
			Mortality	No infarct		
Fig. 5	Ctrl	11	1	3	7	21.6
	cKO	16	2	4	10	21.7
	R-Rosco	7	1	0	6	21.4

### TTC staining and measurement of brain ischemic spreading

Mice were euthanized 24-h post-reperfusion. The brain was removed from the skull and 2 mm coronal sections were made from the olfactory bulb to the cerebellum. One rostral section and one caudal section were stained with 1% TTC (Sigma, St. Louis, MO, USA) (Supplementary Figure 3). The stained sections were captured with a digital camera (Leica, 20×) and the quantification of infarct area of each brain was measured using image analysis software, ImageJ (Java; Wayne Rasband, National Institute of mental Health, Bethesda, MD, USA).

### Neuroscore scale

Following MCAO, mice were tested for neurological deficits at 24-h post-reperfusion and scored on a 5-point scale as previously described^39^: 0, mice behave normally with no detectable neurological deficits (normal); 1, mice fail to extend the right forepaw and show inconsistent rotation movement when hanged by the tail (mild); 2, mice circle to the contralateral side when pushed by the tail (moderate); 3, mice show spontaneous rotation movement and fall to the right (severe); 4, mice show barreling movement and cannot walk spontaneously; 5, mice are moribund and have depressed level of consciousness (very severe).

### Statistical analysis

All quantifications were done by a researcher blind to the experimental conditions. All statistical analyses were performed using Prism 5 (GraphPad software). All data were presented as the mean ± SEM, and significance was determined by Student’s *t*-test or one-way analysis of variance (ANOVA) with Bonferroni’s post hoc to compare differences between groups. All tests were considered statistically significant at *p* < 0.05.

## Electronic supplementary material

Supplementary data(DOCX 1718 kb)

## References

[1] Roth JM (2011). Recombinant tissue plasminogen activator for the treatment of acute ischemic stroke. Proc. (Bayl. Univ. Med. Cent.).

[2] Dirnagl U (1999). Pathobiology of ischaemic stroke: an integrated view. Trends Neurosci..

[3] Rashidian J, Iyirhiaro GO, Park DS (1772). Cell cycle machinery and stroke. Biochim. Biophys. Acta - Mol. Basis Dis..

[4] Frade JM, Ovejero-Benito MC (2015). Neuronal cell cycle: the neuron itself and its circumstances. Cell Cycle.

[5] Castedo M, Perfettini JL, Roumier T, Kroemer G (2002). Cyclin-dependent kinase-1: linking apoptosis to cell cycle and mitotic catastrophe. Cell. Death. Differ..

[6] Wen Y (2004). Transient cerebral ischemia induces aberrant neuronal cell cycle re-entry and Alzheimer’s disease-like tauopathy in female rats. J. Biol. Chem..

[7] Meijer L (1997). Biochemical and cellular effects of roscovitine, a potent and selective inhibitor of the cyclin-dependent kinasescdc2, cdk2 and cdk5. Eur. J. Biochem..

[8] Rashidian J (2005). Multiple cyclin-dependent kinases signals are critical mediators of ischemia/hypoxic neuronal death in vitro and in vivo. Proc. Natl. Acad. Sci. USA.

[9] Katchanov J (2001). Mild cerebral ischemia induces loss of cyclin-dependent kinase inhibitors and activation of cell cycle machinery before delayed neuronal cell death. J. Neurosci..

[10] Gendron TF (2001). Attenuation of neurotoxicity in cortical cultures and hippocampal slices from E2F1 knockout mice. J. Neurochem..

[11] Wen Y, Yang S, Liu R, Simpkins JW (2005). Cell-cycle regulators are involved in transient cerebral ischemia induced neuronal apoptosis in female rats. FEBS Lett..

[12] Chen B, Wang W (2008). The expression of cyclins in neurons of rats after focal cerebral ischemia. J. Huazhong Univ. Sci. Technol. Med Sci..

[13] Kuan CY (2004). Hypoxia-ischemia induces DNA synthesis without cell proliferation in dying neurons in adult rodent brain. J. Neurosci..

[14] Love S (2003). Neuronal expression of cell cycle-related proteins after brain ischaemia in man. Neurosci. Lett..

[15] Meijer L (2016). Modulating innate and adaptive immunity by (R)-roscovitine: potential therapeutic opportunity in cystic fibrosis. J. Innate Immun..

[16] Rousselet, E. et al. Sustained (S)-roscovitinedelivery promotes neuroprotection associated with functional recovery and decrease in brain edema in a randomized blind focal cerebral ischemia study. *J. Cereb. Blood Flow Metab.* (2017) 10.1177/0271678X17712163. 10.1177/0271678X17712163 PMC5998998 28569655

[17] Menn B (2010). Delayed treatment with systemic (s)-roscovitine provides neuroprotection and inhibits in vivo CDK5 activity increase in animal stroke models. PLoS. ONE..

[18] Gutiérrez-Vargas JA, Moreno H, Cardona-Gómez GP (2017). Targeting CDK5 post-stroke provides long-term neuroprotection and rescues synaptic plasticity. J. Cereb. Blood Flow Metab..

[19] Meyer DA (2014). Ischemic stroke injury is mediated by aberrant Cdk5. J. Neurosci..

[20] Liu SL (2016). The role of Cdk5 in Alzheimer’s disease. Mol. Neurobiol..

[21] Sahara S, Yanagawa Y, O’Leary DDM, Stevens CF (2012). The fraction of cortical GABAergic neurons is constant from near the start of cortical neurogenesis to adulthood. J. Neurosci..

[22] Delehouzé C (2014). CDK/CK1 inhibitors roscovitine and CR8 downregulate amplified MYCN in neuroblastoma cells. Oncogene.

[23] Shah K, Lahiri DK (2014). Cdk5 activity in the brain - multiple paths of regulation. J. Cell. Sci..

[24] Odajima J (2011). Cyclin E constrains Cdk5 activity to regulate synaptic plasticity and memory formation. Dev. Cell..

[25] Slevin M, Krupinski J (2009). Cyclin-dependent kinase-5 targeting for ischaemic stroke. Curr. Opin. Pharmacol..

[26] Sundaram JR (2013). Specific inhibition of p25/Cdk5 activity by the Cdk5 inhibitory peptide reduces neurodegeneration in vivo. J. Neurosci..

[27] Konishi Y, Lehtinen M, Donovan N, Bonni A (2002). Cdc2 phosphorylation of BAD links the cell cycle to the cell death machinery. Mol. Cell..

[28] Terrano DT, Upreti M, Chambers TC (2010). Cyclin-dependent kinase 1-mediated Bcl-xL/Bcl-2 phosphorylation acts as a functional link coupling mitotic arrest and apoptosis. Mol. Cell. Biol..

[29] Harley ME, Allan LA, Sanderson HS, Clarke PR (2010). Phosphorylation of Mcl-1 by CDK1-cyclin B1 initiates its Cdc20-dependent destruction during mitotic arrest. EMBO. J..

[30] Bettayeb K (2010). CDK inhibitors roscovitine and CR8 trigger Mcl-1 down-regulation and apoptotic cell death in neuroblastoma cells. Genes Cancer.

[31] Yuan Z (2008). Activation of FOXO1 by Cdk1 in cycling cells and post-mitotic neurons. Science.

[32] Piedrahita D (2010). Silencing of CDK5 reduces neurofibrillary tangles in transgenic alzheimer’s mice. J. Neurosci..

[33] Smith PD (2003). Cyclin-dependent kinase 5 is a mediator of dopaminergic neuron loss in a mouse model of Parkinson’s disease. Proc. Natl. Acad. Sci. USA.

[34] Wu J (2012). Inhibition of E2F1/CDK1 pathway attenuates neuronal apoptosis in vitro and confers neuroprotection after spinal cord injury in vivo. PLoS. ONE..

[35] Pei JJ (2002). Up-regulation of cell division cycle (cdc) 2 kinase in neurons with early stage Alzheimer’s disease neurofibrillary degeneration. Acta Neuropathol..

[36] Diril MK (2012). Cyclin-dependent kinase 1 (Cdk1) is essential for cell division and suppression of DNA re-replication but not for liver regeneration. Proc. Natl. Acad. Sci. USA.

[37] Goebbels S (2006). Genetic targeting of principal neurons in neocortex and hippocampus of NEX-Cre mice. Genesis.

[38] Liu F, Schafer DP, McCullough LD (2009). TTC, Fluoro-Jade B and NeuN staining confirm evolving phases of infarction induced by middle cerebral artery occlusion. J. Neurosci. Methods.

[39] Rousselet E, Kriz J, Seidah NG (2012). Mouse model of itraluminal MCAO: cerebral infarct evaluation by Cresyl violet staining. J. Vis. Exp..

