# Supplementary material for: Genetic and pharmacological inhibition of Cdk1 provides neuroprotection towards ischemic neuronal death
Source: Cell Death Discov. 2018 Mar 16;4:43. doi: 10.1038/s41420-018-0044-7 (PMC5856839; doi:10.1038/s41420-018-0044-7)

**Supplementary data**

**Genetic and pharmacological inhibition of Cdk1 provides neuroprotection towards ischemic neuronal death**

Quentin Marlier^1^, Florian Jibassia^1^, Sébastien Verteneuil^1^, Jérôme Linden^3^, Philipp Kaldis^4,5^ Laurent Meijer^6^ Laurent Nguyen^2^ ], Renaud Vandenbosch^1,7^ and Brigitte Malgrange^1,7,*^

^1^Laboratory of Developmental Neurobiology and ^2^Molecular Regulation of Neurogenesis, GIGA-Neurosciences, University of Liège, C.H.U. B36, 4000 Liège, Belgium

^3^University of Liege, Department of Psychology, B32, 4000 Liège, Belgium

^4^Institute of Molecular and Cell Biology (IMCB), A*STAR (Agency for Science, Technology and Research), 61 Biopolis Drive, Proteos#3-09, Singapore 138673, Republic of Singapore

^5^National University of Singapore (NUS), Department of Biochemistry, Singapore 117597, Republic of Singapore

^6^ ManRos Therapeutics, Centre de Perharidy, 29680 Roscoff, France

^7^ = co-last authors

***Corresponding author:**

Brigitte Malgrange, PhD

Laboratory of Developmental Neurobiology, GIGA-Neurosciences, University of Liège

Quartier Hôpital, Avenue Hippocrate 15, B36 +1, 4000 Liège, Belgium

e-mail: [bmalgrange@ulg.ac.be](mailto:bmalgrange@ulg.ac.be)

**Running title: Neuroprotection by Cdk1 inhibition in ischemia**

**Key words: OGD; ischemic stroke; neuroprotection; R-Roscovitine; Cdk1; cell cycle**

**Supplementary Figures Legends**

**Supplementary Figure 1: Cdk1 is dispensable for physiologic cortical neurons**

(**A**) Representative pictures of Ctrl and Cdk1-cKO brains. Scale bar=1cm. Quantitative results for Ctrl and Cdk1-cKO brain weight (data represent means ± SEM; *n*=4-5; P>0,5; student’s *t* test). (**B**) Cresyl violet and NeuN staining showing rostral cortical thickness respectively as well as the number of neuronal cells (data represents means ± SEM; *n*=4; P>0,5; student’s *t* test). Scale bars, 1mm-20µm. Quantitative results showing no differences in rostral cortical thickness respectively as well as in the number of neuronal cells (data represents means ± SEM; *n*=4; P>0,5; student’s *t* test). (**C**) Cresyl violet and NeuN staining showing medial cortical thickness respectively as well as the number of neuronal cells (data represents means ± SEM; *n*=4; P>0,5; student’s *t* test). Scale bars, 1mm-20µm. Quantitative results showing no differences in medial cortical thickness respectively as well as in the number of neuronal cells (data represents means ± SEM; *n*=4; P>0,5; student’s *t* test).

**Supplementary Figure 2: Culture conditions do not lead to significant glial contamination**

(**A**) Representative confocal images illustrating the virtual absence glial contamination in primary cortical neurons culture either in normoxic or OGD condition 24h following OGD or normoxia. (**B**) Quantitative results for glial contamination m. represents the percentage of double labeled DAPI+/GFAP+ cells per field (318,2µm x 318,2µm), scale bar= 50µ

**Supplementary Figure 3: Selection of 2mm thick rostral and caudal slices**

Scheme of adult mouse brain slicer matrix. 2 mm thick slices (5 slices in total) have been collected from the beginning to the end of the cortex. A rostral slice (2^nd^) and a caudal slice (4^st^) have been selected to be stained with TTC.

**Supplementary Figures**

**
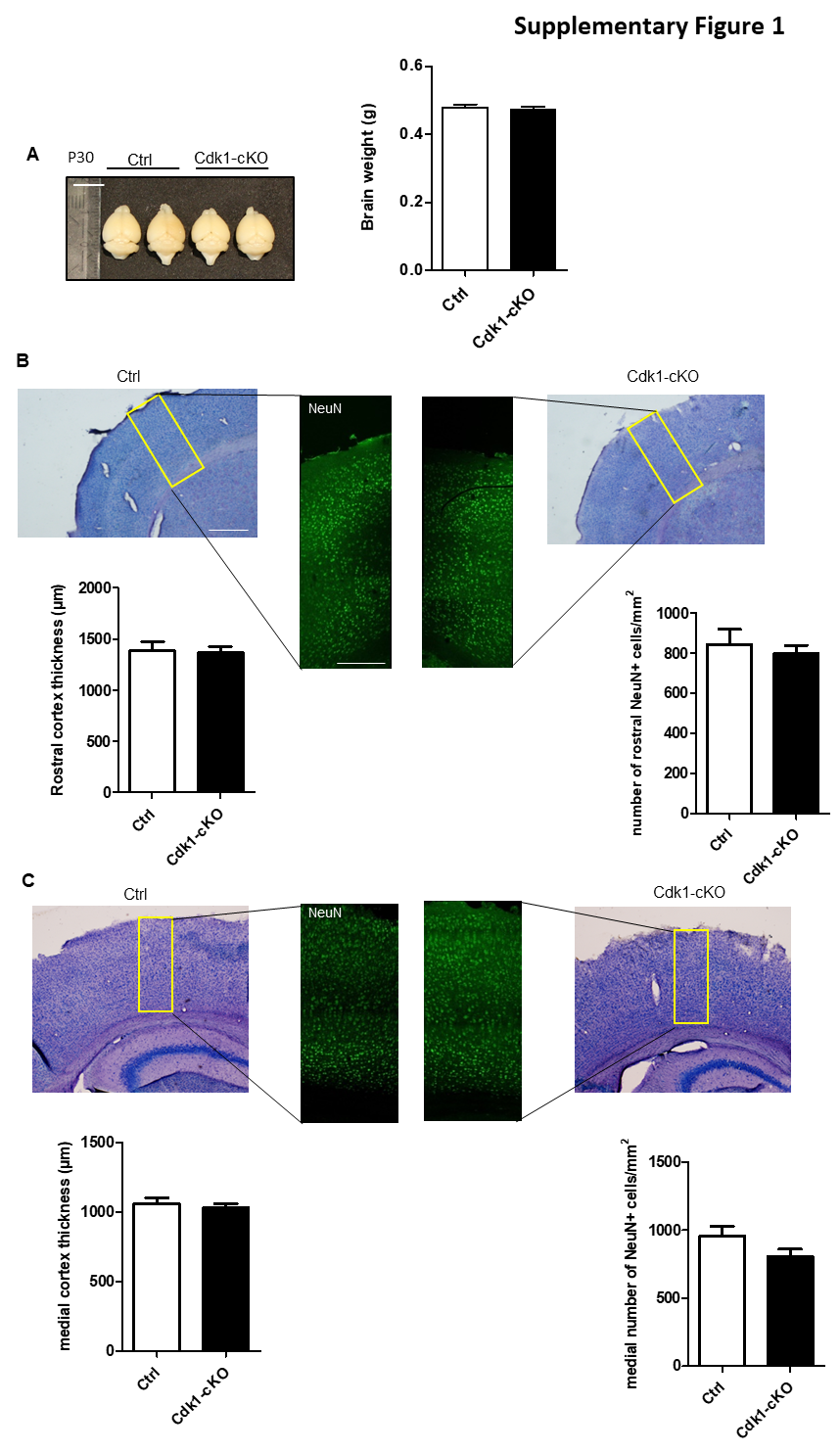
**


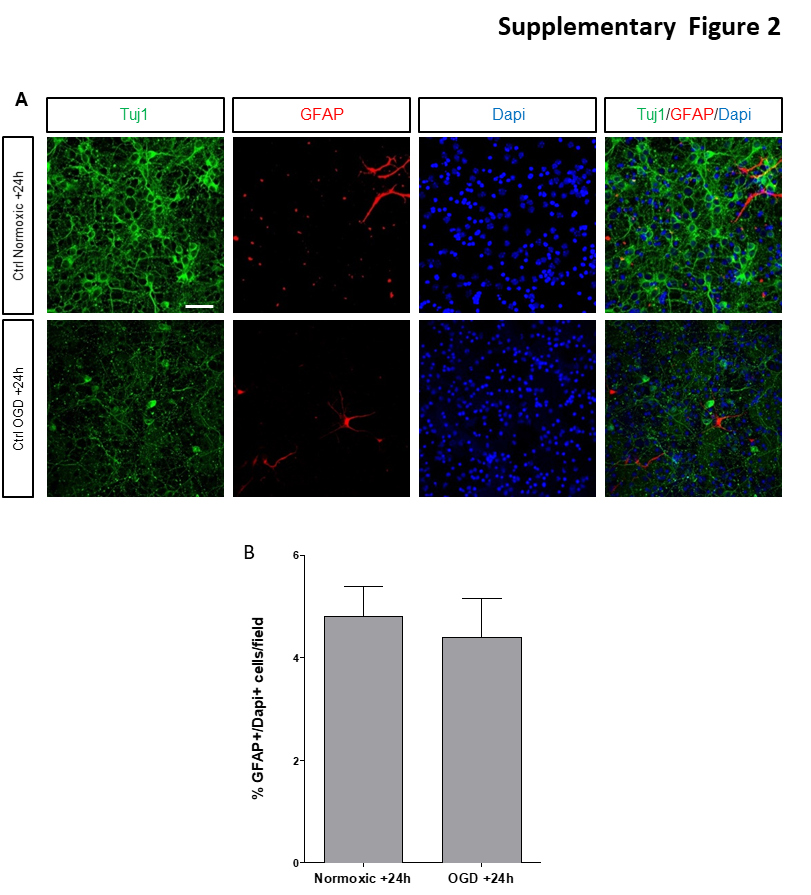


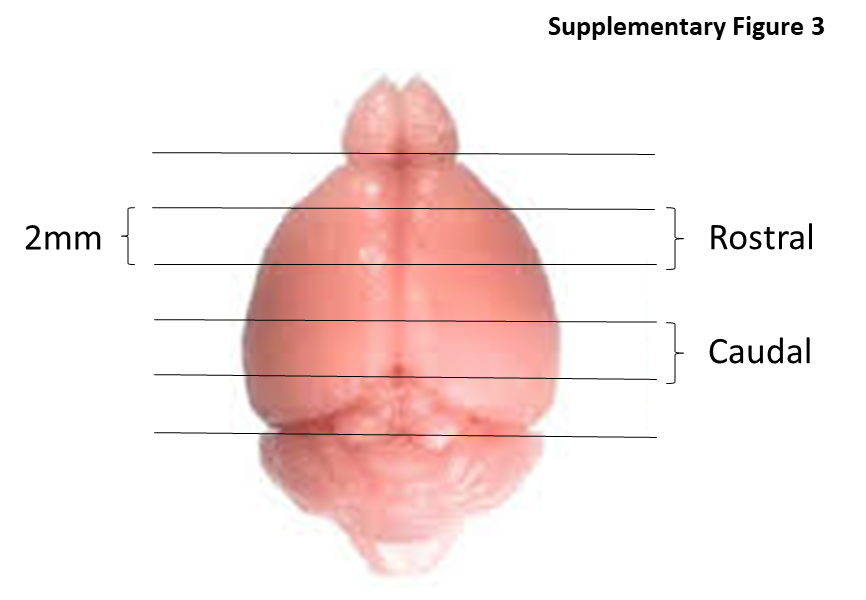

Supplement: Supplementary file 1 — Supplementary data(DOCX 1718 kb) [file 41420_2018_44_MOESM1_ESM.docx]
